# Tris­(nitrato-κ^2^ *O*,*O*′)bis[4,4,5,5-tetra­methyl-2-(pyridin-2-yl-κ*N*)imidazoline-1-oxyl 3-oxide-κ*O*]holmium(III)

**DOI:** 10.1107/S1600536812012445

**Published:** 2012-04-06

**Authors:** Dong-Jiao Li

**Affiliations:** a Chemical Institute, Linyi University, Linyi Shandong 276005, People’s Republic of China

## Abstract

In the title compound, [Ho(NO_3_)_3_(C_12_H_16_N_3_O_2_)_2_], the Ho^III^ ion is ten-coordinated in a distorted bicapped square-anti­prismatic environment by two *N*,*O*-bidentate nitronyl nitroxide radical ligands and three *O*,*O*′-bidentate nitrate anions. Complex mol­ecules are connected by C—H⋯O hydrogen bonds into a three-dimensional network.

## Related literature

For background on the use of rare earth complexes with nitroxide radicals in coordination chemistry, see: Sutter *et al.* (1998 ▶); Kahn *et al.* (2000 ▶); Lescop *et al.* (2000 ▶). For related complexes reported by our group, see: Li *et al.* (2005 ▶); Li, Gao & Liao (2004 ▶); Li, Wang & Liao (2004 ▶).
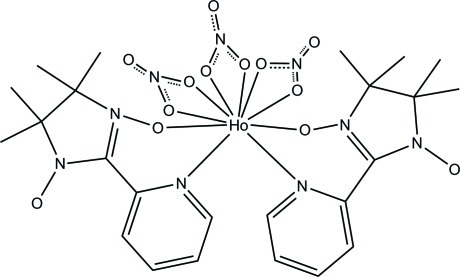


## Experimental

### 

#### Crystal data

[Ho(NO_3_)_3_(C_12_H_16_N_3_O_2_)_2_]
*M* *_r_* = 819.52
Monoclinic, 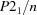

*a* = 12.2627 (10) Å
*b* = 11.1044 (8) Å
*c* = 23.2861 (17) Å
β = 98.391 (2)°
*V* = 3136.9 (4) Å^3^
*Z* = 4
Mo *K*α radiation
μ = 2.60 mm^−1^
*T* = 293 K
0.22 × 0.16 × 0.12 mm

#### Data collection

Bruker APEXII CCD diffractometer
Absorption correction: multi-scan (*SADABS*; Sheldrick, 2004 ▶) *T*_min_ = 0.599, *T*_max_ = 0.746
17689 measured reflections
5530 independent reflections
4895 reflections with *I* > 2σ(*I*)
*R*_int_ = 0.047

#### Refinement

*R*[*F* ^2^ > 2σ(*F* ^2^)] = 0.044
*wR*(*F* ^2^) = 0.061
*S* = 1.25
5530 reflections
432 parameters
H-atom parameters constrained
Δρ_max_ = 0.51 e Å^−3^
Δρ_min_ = −0.87 e Å^−3^

### 

Data collection: *APEX2* (Bruker, 2004 ▶); cell refinement: *SAINT* (Bruker, 2004 ▶); data reduction: *SAINT*; program(s) used to solve structure: *SHELXS97* (Sheldrick, 2008 ▶); program(s) used to refine structure: *SHELXL97* (Sheldrick, 2008 ▶); molecular graphics: *SHELXTL* (Sheldrick, 2008 ▶); software used to prepare material for publication: *SHELXTL*.

## Supplementary Material

Crystal structure: contains datablock(s) I, global. DOI: 10.1107/S1600536812012445/is5078sup1.cif

Structure factors: contains datablock(s) I. DOI: 10.1107/S1600536812012445/is5078Isup2.hkl

Additional supplementary materials:  crystallographic information; 3D view; checkCIF report

## Figures and Tables

**Table 1:** Hydrogen-bond geometry (Å, °)

*D*—H⋯*A*	*D*—H	H⋯*A*	*D*⋯*A*	*D*—H⋯*A*
C8—H8⋯O11^i^	0.93	2.42	3.053 (5)	126
C11—H11*B*⋯O13^ii^	0.96	2.54	3.460 (6)	161
C18—H18⋯O4^iii^	0.93	2.56	3.450 (6)	160
C20—H20⋯O7^iv^	0.93	2.37	3.193 (5)	147
C22—H22*C*⋯O13^i^	0.96	2.53	3.239 (6)	131
C24—H24*B*⋯O10^v^	0.96	2.40	3.324 (6)	162

Symmetry codes: (i) 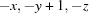; (ii) 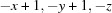; (iii) 
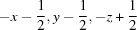
; (iv) 
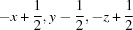
; (v) 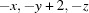.

**Fig. 1.**
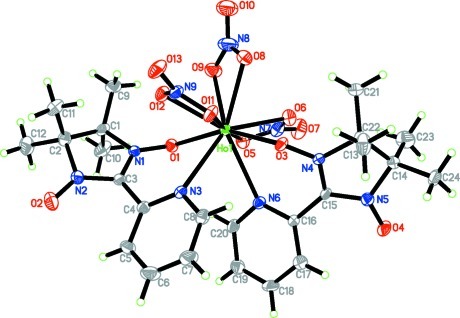
The molecular structure of the title compound drawn with 30% ellipsoidal probability.

**Fig. 2.**
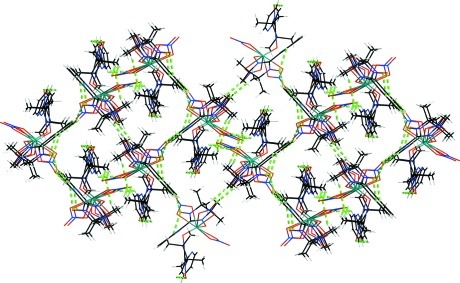
A packing diagram of the title compound, showing a three-dimensional network formed by intermolecular C—H···O hydrogen bonds.

## supplementary crystallographic information

### Comment

As a continuation of our work on the complexes adopting nitroxide radicals as the ligand, the title new Ho complex is reported. In this compound, the metal ion is ten-coordinated by two radicals and three nitrate anions (Fig. 1). The radical behaves as a bidentate chelating ligand through one oxygen of nitronyl nitroxide and one nitrogen of pyridyl, while the other oxygen of nitronyl nitroxide remains uncoordinated. The coordination sphere of the Ho atom is completed by the η^2^-coordination of three NO_3_^-^ anions. The complexes are further connected by C—H···O hydrogen bonds (Table 1) into a three-dimensional framework, as shown in Fig. 2.

### Experimental

The compound was synthesized by the following procedure. Ho(NO_3_)_3_.6H_2_O (0.046 g, 0.2 mmol) and NIT2Py (0.047 g, 0.2 mmol) were dissolved in 10ml of anhydrous THF. The mixture was stirred at room temperature for four hours and then filtered. The dark brown filtrate was allowed to stand in the dark for one week. Dark brown crystals were obtained.

### Refinement

H atoms were positioned geometrically (C—H = 0.93–0.96 Å) and refined using a riding model, with *U*_iso_(H) = 1.2*U*_eq_(C) and 1.5*U*_eq_(C_methyl_).

### Crystal data

**Table:**

[Ho(NO_3_)_3_(C_12_H_16_N_3_O_2_)_2_]	*F*(000) = 1640
*M**_r_* = 819.52	*D*_x_ = 1.735 Mg m^−^^3^
Monoclinic, *P*2_1_/*n*	Mo *K*α radiation, λ = 0.71075 Å
Hall symbol: -P 2yn	Cell parameters from 2811 reflections
*a* = 12.2627 (10) Å	θ = 2.5–28.8°
*b* = 11.1044 (8) Å	µ = 2.60 mm^−^^1^
*c* = 23.2861 (17) Å	*T* = 293 K
β = 98.391 (2)°	Block, dark brown
*V* = 3136.9 (4) Å^3^	0.22 × 0.16 × 0.12 mm
*Z* = 4	

### Data collection

**Table:**

Bruker APEXII CCD diffractometer	5530 independent reflections
Radiation source: fine-focus sealed tube	4895 reflections with *I* > 2σ(*I*)
Graphite monochromator	*R*_int_ = 0.047
φ and ω scans	θ_max_ = 25.0°, θ_min_ = 3.2°
Absorption correction: multi-scan (*SADABS*; Sheldrick, 2004)	*h* = −14→14
*T*_min_ = 0.599, *T*_max_ = 0.746	*k* = −10→13
17689 measured reflections	*l* = −27→27

### Refinement

**Table:**

Refinement on *F*^2^	Primary atom site location: structure-invariant direct methods
Least-squares matrix: full	Secondary atom site location: difference Fourier map
*R*[*F*^2^ > 2σ(*F*^2^)] = 0.044	Hydrogen site location: inferred from neighbouring sites
*wR*(*F*^2^) = 0.061	H-atom parameters constrained
*S* = 1.25	*w* = 1/[σ^2^(*F*_o_^2^) + (0.0167*P*)^2^] where *P* = (*F*_o_^2^ + 2*F*_c_^2^)/3
5530 reflections	(Δ/σ)_max_ < 0.001
432 parameters	Δρ_max_ = 0.51 e Å^−^^3^
0 restraints	Δρ_min_ = −0.87 e Å^−^^3^

### Special details

**Table:**

Geometry. All e.s.d.'s (except the e.s.d. in the dihedral angle between two l.s. planes) are estimated using the full covariance matrix. The cell e.s.d.'s are taken into account individually in the estimation of e.s.d.'s in distances, angles and torsion angles; correlations between e.s.d.'s in cell parameters are only used when they are defined by crystal symmetry. An approximate (isotropic) treatment of cell e.s.d.'s is used for estimating e.s.d.'s involving l.s. planes.
Refinement. Refinement of *F*^2^ against ALL reflections. The weighted *R*-factor *wR* and goodness of fit *S* are based on *F*^2^, conventional *R*-factors *R* are based on *F*, with *F* set to zero for negative *F*^2^. The threshold expression of *F*^2^ > σ(*F*^2^) is used only for calculating *R*-factors(gt) *etc*. and is not relevant to the choice of reflections for refinement. *R*-factors based on *F*^2^ are statistically about twice as large as those based on *F*, and *R*- factors based on ALL data will be even larger.

### Fractional atomic coordinates and isotropic or equivalent isotropic displacement parameters (Å^2^)

**Table:**

	*x*	*y*	*z*	*U*_iso_*/*U*_eq_	
Ho1	0.179214 (16)	0.661468 (16)	0.104145 (7)	0.02420 (7)	
O1	0.3290 (2)	0.5763 (2)	0.16176 (10)	0.0300 (7)	
O2	0.4048 (3)	0.1762 (3)	0.12909 (15)	0.0620 (10)	
O3	−0.0085 (2)	0.6824 (2)	0.06574 (10)	0.0292 (7)	
O4	−0.2481 (3)	0.7880 (3)	0.19251 (11)	0.0419 (8)	
O5	0.2368 (3)	0.7701 (3)	0.19958 (12)	0.0409 (8)	
O6	0.1138 (3)	0.8544 (2)	0.13632 (12)	0.0399 (8)	
O7	0.1635 (3)	0.9416 (3)	0.21931 (13)	0.0589 (10)	
O8	0.1982 (3)	0.8043 (3)	0.02635 (12)	0.0448 (9)	
O9	0.3316 (3)	0.7986 (3)	0.09759 (13)	0.0473 (9)	
O10	0.3446 (4)	0.9135 (4)	0.02325 (19)	0.1036 (16)	
O11	0.1190 (2)	0.5707 (3)	0.00788 (11)	0.0368 (8)	
O12	0.2943 (2)	0.5667 (3)	0.03594 (11)	0.0367 (8)	
O13	0.2278 (3)	0.5218 (3)	−0.05394 (11)	0.0496 (9)	
N1	0.3807 (3)	0.4780 (3)	0.15077 (12)	0.0260 (8)	
N2	0.4172 (3)	0.2890 (3)	0.13669 (14)	0.0356 (9)	
N3	0.1487 (3)	0.4267 (3)	0.11502 (12)	0.0260 (8)	
N4	−0.0860 (3)	0.7361 (3)	0.08828 (12)	0.0222 (8)	
N5	−0.2006 (3)	0.7847 (3)	0.14709 (13)	0.0297 (9)	
N6	0.0492 (3)	0.6136 (3)	0.18514 (12)	0.0274 (8)	
N7	0.1718 (3)	0.8572 (3)	0.18637 (15)	0.0381 (10)	
N8	0.2937 (4)	0.8419 (4)	0.04853 (19)	0.0535 (11)	
N9	0.2134 (3)	0.5520 (3)	−0.00485 (14)	0.0309 (9)	
C1	0.5041 (4)	0.4773 (4)	0.15474 (16)	0.0336 (11)	
C2	0.5205 (4)	0.3531 (4)	0.12639 (18)	0.0392 (12)	
C3	0.3361 (4)	0.3693 (4)	0.14364 (16)	0.0272 (10)	
C4	0.2199 (4)	0.3408 (4)	0.13886 (15)	0.0269 (10)	
C5	0.1855 (4)	0.2282 (4)	0.15475 (17)	0.0375 (12)	
H5	0.2361	0.1725	0.1726	0.045*	
C6	0.0752 (5)	0.2005 (4)	0.1436 (2)	0.0530 (14)	
H6	0.0501	0.1258	0.1542	0.064*	
C7	0.0022 (4)	0.2843 (4)	0.11667 (19)	0.0470 (13)	
H7	−0.0724	0.2665	0.1075	0.056*	
C8	0.0427 (4)	0.3952 (4)	0.10373 (17)	0.0349 (11)	
H8	−0.0071	0.4518	0.0860	0.042*	
C9	0.5416 (4)	0.5855 (4)	0.12275 (19)	0.0467 (13)	
H9A	0.5027	0.5872	0.0839	0.070*	
H9B	0.6193	0.5798	0.1216	0.070*	
H9C	0.5263	0.6580	0.1426	0.070*	
C10	0.5497 (4)	0.4825 (4)	0.21934 (17)	0.0529 (14)	
H10A	0.5257	0.5556	0.2357	0.079*	
H10B	0.6288	0.4807	0.2242	0.079*	
H10C	0.5232	0.4146	0.2387	0.079*	
C11	0.5140 (4)	0.3573 (4)	0.05990 (18)	0.0517 (14)	
H11A	0.5135	0.2767	0.0449	0.077*	
H11B	0.5768	0.3998	0.0499	0.077*	
H11C	0.4477	0.3980	0.0434	0.077*	
C12	0.6227 (4)	0.2824 (5)	0.1523 (2)	0.0628 (16)	
H12A	0.6217	0.2704	0.1930	0.094*	
H12B	0.6877	0.3266	0.1468	0.094*	
H12C	0.6231	0.2056	0.1333	0.094*	
C13	−0.1611 (3)	0.8248 (4)	0.05230 (15)	0.0266 (10)	
C14	−0.2272 (4)	0.8757 (3)	0.09915 (16)	0.0279 (10)	
C15	−0.1146 (3)	0.7139 (3)	0.13957 (15)	0.0239 (10)	
C16	−0.0604 (4)	0.6276 (3)	0.18221 (16)	0.0256 (10)	
C17	−0.1219 (4)	0.5649 (4)	0.21723 (17)	0.0391 (12)	
H17	−0.1977	0.5764	0.2139	0.047*	
C18	−0.0689 (4)	0.4842 (4)	0.25749 (18)	0.0503 (14)	
H18	−0.1091	0.4376	0.2801	0.060*	
C19	0.0436 (4)	0.4743 (4)	0.26343 (17)	0.0444 (13)	
H19	0.0815	0.4243	0.2915	0.053*	
C20	0.0991 (4)	0.5405 (4)	0.22672 (16)	0.0337 (11)	
H20	0.1755	0.5341	0.2311	0.040*	
C21	−0.0891 (4)	0.9138 (4)	0.02590 (19)	0.0496 (14)	
H21A	−0.0373	0.9493	0.0561	0.074*	
H21B	−0.1343	0.9758	0.0060	0.074*	
H21C	−0.0499	0.8728	−0.0011	0.074*	
C22	−0.2297 (4)	0.7515 (4)	0.00520 (17)	0.0478 (14)	
H22A	−0.1819	0.7113	−0.0178	0.072*	
H22B	−0.2787	0.8040	−0.0191	0.072*	
H22C	−0.2720	0.6928	0.0227	0.072*	
C23	−0.1829 (4)	0.9964 (4)	0.12467 (19)	0.0556 (15)	
H23A	−0.2192	1.0168	0.1572	0.083*	
H23B	−0.1967	1.0580	0.0956	0.083*	
H23C	−0.1050	0.9899	0.1372	0.083*	
C24	−0.3505 (4)	0.8831 (4)	0.08100 (19)	0.0468 (13)	
H24A	−0.3787	0.8048	0.0693	0.070*	
H24B	−0.3665	0.9382	0.0491	0.070*	
H24C	−0.3848	0.9112	0.1131	0.070*	

### Atomic displacement parameters (Å^2^)

**Table:**

	*U*^11^	*U*^22^	*U*^33^	*U*^12^	*U*^13^	*U*^23^
Ho1	0.02112 (12)	0.02742 (11)	0.02412 (10)	0.00062 (10)	0.00349 (7)	0.00204 (10)
O1	0.026 (2)	0.0304 (17)	0.0323 (15)	0.0016 (14)	−0.0011 (13)	0.0009 (13)
O2	0.058 (3)	0.035 (2)	0.097 (3)	0.0114 (19)	0.022 (2)	0.0032 (19)
O3	0.0251 (19)	0.0381 (18)	0.0255 (14)	0.0006 (14)	0.0078 (13)	−0.0020 (13)
O4	0.040 (2)	0.059 (2)	0.0308 (16)	0.0138 (17)	0.0180 (15)	0.0043 (15)
O5	0.036 (2)	0.0411 (19)	0.0423 (18)	0.0046 (17)	−0.0046 (15)	−0.0030 (15)
O6	0.039 (2)	0.0376 (19)	0.0405 (17)	0.0011 (16)	−0.0021 (15)	−0.0058 (15)
O7	0.055 (3)	0.057 (2)	0.064 (2)	−0.0006 (19)	0.0049 (18)	−0.0334 (19)
O8	0.040 (3)	0.046 (2)	0.0486 (19)	0.0036 (17)	0.0078 (17)	0.0151 (16)
O9	0.043 (2)	0.046 (2)	0.0515 (19)	−0.0085 (16)	0.0003 (17)	0.0174 (16)
O10	0.086 (4)	0.104 (3)	0.123 (3)	−0.028 (3)	0.024 (3)	0.071 (3)
O11	0.023 (2)	0.058 (2)	0.0302 (15)	0.0053 (16)	0.0062 (14)	−0.0034 (14)
O12	0.023 (2)	0.053 (2)	0.0328 (16)	0.0063 (15)	0.0007 (14)	0.0013 (14)
O13	0.048 (2)	0.073 (2)	0.0312 (16)	0.0028 (19)	0.0177 (15)	−0.0082 (17)
N1	0.021 (2)	0.031 (2)	0.0260 (17)	0.0010 (18)	0.0028 (15)	0.0086 (16)
N2	0.028 (3)	0.033 (2)	0.047 (2)	0.0052 (19)	0.0068 (18)	0.0088 (18)
N3	0.021 (2)	0.031 (2)	0.0264 (18)	−0.0020 (17)	0.0049 (15)	−0.0031 (15)
N4	0.020 (2)	0.0232 (19)	0.0239 (17)	−0.0016 (16)	0.0050 (15)	−0.0010 (15)
N5	0.028 (2)	0.037 (2)	0.0248 (18)	0.0052 (18)	0.0062 (16)	0.0020 (16)
N6	0.026 (2)	0.034 (2)	0.0224 (17)	0.0016 (17)	0.0043 (16)	0.0022 (15)
N7	0.030 (3)	0.043 (3)	0.042 (2)	−0.009 (2)	0.0046 (19)	−0.012 (2)
N8	0.047 (3)	0.047 (3)	0.070 (3)	0.000 (3)	0.020 (3)	0.019 (3)
N9	0.025 (3)	0.040 (2)	0.029 (2)	−0.0007 (18)	0.0073 (18)	0.0022 (17)
C1	0.016 (3)	0.047 (3)	0.037 (2)	0.001 (2)	0.0040 (19)	0.008 (2)
C2	0.023 (3)	0.047 (3)	0.048 (3)	0.005 (2)	0.005 (2)	0.009 (2)
C3	0.023 (3)	0.031 (3)	0.027 (2)	0.002 (2)	0.0033 (19)	0.0065 (18)
C4	0.027 (3)	0.031 (2)	0.0221 (19)	0.001 (2)	0.0027 (18)	0.003 (2)
C5	0.031 (3)	0.037 (3)	0.044 (3)	−0.002 (2)	0.001 (2)	0.013 (2)
C6	0.050 (4)	0.042 (3)	0.066 (3)	−0.016 (3)	0.006 (3)	0.012 (3)
C7	0.032 (3)	0.050 (3)	0.059 (3)	−0.016 (3)	0.003 (3)	0.001 (3)
C8	0.028 (3)	0.039 (3)	0.036 (2)	−0.001 (2)	0.000 (2)	−0.003 (2)
C9	0.031 (3)	0.050 (3)	0.061 (3)	−0.011 (3)	0.010 (2)	0.010 (3)
C10	0.027 (3)	0.088 (4)	0.041 (3)	−0.003 (3)	−0.003 (2)	0.002 (3)
C11	0.041 (4)	0.066 (4)	0.052 (3)	−0.001 (3)	0.020 (2)	−0.001 (3)
C12	0.030 (4)	0.066 (4)	0.090 (4)	0.016 (3)	0.003 (3)	0.014 (3)
C13	0.022 (3)	0.032 (2)	0.027 (2)	0.008 (2)	0.0055 (18)	0.0047 (19)
C14	0.028 (3)	0.024 (2)	0.033 (2)	0.0052 (19)	0.007 (2)	0.0033 (18)
C15	0.018 (3)	0.029 (2)	0.025 (2)	0.000 (2)	0.0060 (18)	0.0012 (18)
C16	0.024 (3)	0.027 (2)	0.027 (2)	0.0002 (19)	0.0057 (19)	−0.0006 (18)
C17	0.020 (3)	0.052 (3)	0.048 (3)	0.004 (2)	0.016 (2)	0.015 (2)
C18	0.050 (4)	0.056 (3)	0.048 (3)	−0.004 (3)	0.019 (3)	0.026 (3)
C19	0.049 (4)	0.050 (3)	0.035 (2)	0.014 (3)	0.009 (2)	0.014 (2)
C20	0.027 (3)	0.046 (3)	0.028 (2)	0.007 (2)	0.004 (2)	0.003 (2)
C21	0.046 (4)	0.046 (3)	0.062 (3)	0.011 (3)	0.029 (3)	0.024 (3)
C22	0.046 (4)	0.060 (3)	0.033 (3)	0.019 (3)	−0.008 (2)	−0.009 (2)
C23	0.072 (5)	0.034 (3)	0.063 (3)	−0.006 (3)	0.018 (3)	−0.012 (3)
C24	0.029 (3)	0.066 (3)	0.047 (3)	0.014 (3)	0.012 (2)	0.016 (3)

### Geometric parameters (Å, º)

**Table:**

Ho1—O1	2.313 (3)	C5—H5	0.9300
Ho1—O3	2.356 (3)	C6—C7	1.378 (6)
Ho1—O9	2.432 (3)	C6—H6	0.9300
Ho1—O6	2.443 (3)	C7—C8	1.377 (6)
Ho1—O8	2.445 (3)	C7—H7	0.9300
Ho1—O11	2.471 (3)	C8—H8	0.9300
Ho1—O12	2.505 (3)	C9—H9A	0.9600
Ho1—O5	2.537 (3)	C9—H9B	0.9600
Ho1—N3	2.651 (3)	C9—H9C	0.9600
Ho1—N6	2.694 (3)	C10—H10A	0.9600
O1—N1	1.305 (4)	C10—H10B	0.9600
O2—N2	1.271 (4)	C10—H10C	0.9600
O3—N4	1.296 (4)	C11—H11A	0.9600
O4—N5	1.280 (4)	C11—H11B	0.9600
O5—N7	1.262 (4)	C11—H11C	0.9600
O6—N7	1.274 (4)	C12—H12A	0.9600
O7—N7	1.225 (4)	C12—H12B	0.9600
O8—N8	1.280 (5)	C12—H12C	0.9600
O9—N8	1.264 (5)	C13—C21	1.515 (6)
O10—N8	1.214 (5)	C13—C22	1.518 (5)
O11—N9	1.253 (4)	C13—C14	1.557 (5)
O12—N9	1.280 (4)	C14—C24	1.512 (6)
O13—N9	1.228 (4)	C14—C23	1.533 (5)
N1—C3	1.325 (5)	C15—C16	1.467 (5)
N1—C1	1.503 (5)	C16—C17	1.378 (5)
N2—O2	1.271 (4)	C17—C18	1.388 (6)
N2—C3	1.363 (5)	C17—H17	0.9300
N2—C2	1.502 (6)	C18—C19	1.370 (6)
N3—C8	1.335 (5)	C18—H18	0.9300
N3—C4	1.356 (5)	C19—C20	1.380 (5)
N4—C15	1.316 (4)	C19—H19	0.9300
N4—C13	1.515 (5)	C20—H20	0.9300
N5—O4	1.280 (4)	C21—H21A	0.9600
N5—C15	1.347 (5)	C21—H21B	0.9600
N5—C14	1.506 (5)	C21—H21C	0.9600
N6—C20	1.341 (5)	C22—H22A	0.9600
N6—C16	1.344 (5)	C22—H22B	0.9600
C1—C9	1.520 (6)	C22—H22C	0.9600
C1—C10	1.527 (5)	C23—H23A	0.9600
C1—C2	1.555 (6)	C23—H23B	0.9600
C2—C12	1.527 (6)	C23—H23C	0.9600
C2—C11	1.539 (6)	C24—H24A	0.9600
C3—C4	1.448 (6)	C24—H24B	0.9600
C4—C5	1.388 (5)	C24—H24C	0.9600
C5—C6	1.374 (6)		
			
O1—Ho1—O3	155.46 (9)	N1—C3—C4	126.3 (4)
O1—Ho1—O9	74.95 (10)	N2—C3—C4	125.1 (4)
O3—Ho1—O9	129.14 (10)	N3—C4—C5	122.7 (4)
O1—Ho1—O6	116.69 (9)	N3—C4—C3	116.5 (4)
O3—Ho1—O6	71.56 (9)	C5—C4—C3	120.7 (4)
O9—Ho1—O6	76.09 (10)	C6—C5—C4	118.7 (4)
O1—Ho1—O8	122.81 (11)	C6—C5—H5	120.6
O3—Ho1—O8	81.26 (10)	C4—C5—H5	120.6
O9—Ho1—O8	52.42 (10)	C5—C6—C7	119.4 (5)
O6—Ho1—O8	74.28 (10)	C5—C6—H6	120.3
O1—Ho1—O11	117.79 (9)	C7—C6—H6	120.3
O3—Ho1—O11	63.23 (9)	C8—C7—C6	118.2 (5)
O9—Ho1—O11	109.23 (10)	C8—C7—H7	120.9
O6—Ho1—O11	124.62 (9)	C6—C7—H7	120.9
O8—Ho1—O11	68.94 (10)	N3—C8—C7	124.3 (4)
O1—Ho1—O12	73.90 (9)	N3—C8—H8	117.9
O3—Ho1—O12	114.22 (9)	C7—C8—H8	117.9
O9—Ho1—O12	73.41 (10)	C1—C9—H9A	109.5
O6—Ho1—O12	143.44 (10)	C1—C9—H9B	109.5
O8—Ho1—O12	71.31 (10)	H9A—C9—H9B	109.5
O11—Ho1—O12	51.30 (9)	C1—C9—H9C	109.5
O1—Ho1—O5	65.83 (10)	H9A—C9—H9C	109.5
O3—Ho1—O5	114.66 (10)	H9B—C9—H9C	109.5
O9—Ho1—O5	68.85 (10)	C1—C10—H10A	109.5
O6—Ho1—O5	51.40 (9)	C1—C10—H10B	109.5
O8—Ho1—O5	107.23 (10)	H10A—C10—H10B	109.5
O12—Ho1—O5	130.15 (10)	C1—C10—H10C	109.5
O1—Ho1—N3	69.74 (10)	H10A—C10—H10C	109.5
O3—Ho1—N3	89.40 (10)	H10B—C10—H10C	109.5
O9—Ho1—N3	137.92 (11)	C2—C11—H11A	109.5
O6—Ho1—N3	140.87 (10)	C2—C11—H11B	109.5
O8—Ho1—N3	137.65 (10)	H11A—C11—H11B	109.5
O11—Ho1—N3	69.98 (9)	C2—C11—H11C	109.5
O12—Ho1—N3	75.40 (10)	H11A—C11—H11C	109.5
O5—Ho1—N3	114.15 (9)	H11B—C11—H11C	109.5
O1—Ho1—N6	90.83 (10)	C2—C12—H12A	109.5
O3—Ho1—N6	68.89 (9)	C2—C12—H12B	109.5
O9—Ho1—N6	134.89 (10)	H12A—C12—H12B	109.5
O6—Ho1—N6	72.78 (10)	C2—C12—H12C	109.5
O8—Ho1—N6	140.96 (11)	H12A—C12—H12C	109.5
O11—Ho1—N6	115.18 (10)	H12B—C12—H12C	109.5
O12—Ho1—N6	143.77 (10)	N4—C13—C21	107.8 (3)
O5—Ho1—N6	66.29 (10)	N4—C13—C22	106.2 (3)
N3—Ho1—N6	68.47 (10)	C21—C13—C22	110.6 (3)
N1—O1—Ho1	126.5 (2)	N4—C13—C14	100.7 (3)
N4—O3—Ho1	128.7 (2)	C21—C13—C14	116.2 (3)
N7—O5—Ho1	93.8 (2)	C22—C13—C14	114.3 (4)
N7—O6—Ho1	97.9 (2)	N5—C14—C24	110.1 (3)
N8—O8—Ho1	95.3 (2)	N5—C14—C23	105.9 (3)
N8—O9—Ho1	96.4 (3)	C24—C14—C23	110.2 (4)
N9—O11—Ho1	96.8 (2)	N5—C14—C13	101.3 (3)
N9—O12—Ho1	94.4 (2)	C24—C14—C13	115.2 (3)
O1—N1—C3	125.7 (4)	C23—C14—C13	113.4 (4)
O1—N1—C1	120.3 (3)	N4—C15—N5	109.0 (3)
C3—N1—C1	113.3 (4)	N4—C15—C16	125.6 (4)
O2—N2—C3	125.9 (4)	N5—C15—C16	125.4 (3)
O2—N2—C3	125.9 (4)	N6—C16—C17	122.8 (4)
O2—N2—C2	122.1 (4)	N6—C16—C15	117.4 (3)
O2—N2—C2	122.1 (4)	C17—C16—C15	119.8 (4)
C3—N2—C2	110.9 (3)	C16—C17—C18	118.9 (4)
C8—N3—C4	116.6 (4)	C16—C17—H17	120.6
C8—N3—Ho1	112.7 (3)	C18—C17—H17	120.6
C4—N3—Ho1	129.7 (3)	C19—C18—C17	119.0 (4)
O3—N4—C15	126.1 (3)	C19—C18—H18	120.5
O3—N4—C13	119.8 (3)	C17—C18—H18	120.5
C15—N4—C13	113.8 (3)	C18—C19—C20	118.4 (4)
O4—N5—C15	125.7 (3)	C18—C19—H19	120.8
O4—N5—C15	125.7 (3)	C20—C19—H19	120.8
O4—N5—C14	121.2 (3)	N6—C20—C19	123.7 (4)
O4—N5—C14	121.2 (3)	N6—C20—H20	118.2
C15—N5—C14	112.6 (3)	C19—C20—H20	118.2
C20—N6—C16	117.0 (3)	C13—C21—H21A	109.5
C20—N6—Ho1	111.7 (3)	C13—C21—H21B	109.5
C16—N6—Ho1	129.1 (2)	H21A—C21—H21B	109.5
O7—N7—O5	122.8 (4)	C13—C21—H21C	109.5
O7—N7—O6	120.3 (4)	H21A—C21—H21C	109.5
O5—N7—O6	116.9 (3)	H21B—C21—H21C	109.5
O10—N8—O9	122.7 (5)	C13—C22—H22A	109.5
O10—N8—O8	121.5 (5)	C13—C22—H22B	109.5
O9—N8—O8	115.8 (4)	H22A—C22—H22B	109.5
O13—N9—O11	121.9 (3)	C13—C22—H22C	109.5
O13—N9—O12	121.6 (4)	H22A—C22—H22C	109.5
O11—N9—O12	116.5 (3)	H22B—C22—H22C	109.5
N1—C1—C9	109.8 (3)	C14—C23—H23A	109.5
N1—C1—C10	106.3 (3)	C14—C23—H23B	109.5
C9—C1—C10	111.0 (4)	H23A—C23—H23B	109.5
N1—C1—C2	99.8 (3)	C14—C23—H23C	109.5
C9—C1—C2	115.2 (4)	H23A—C23—H23C	109.5
C10—C1—C2	113.6 (4)	H23B—C23—H23C	109.5
N2—C2—C12	110.9 (4)	C14—C24—H24A	109.5
N2—C2—C11	104.7 (3)	C14—C24—H24B	109.5
C12—C2—C11	109.4 (4)	H24A—C24—H24B	109.5
N2—C2—C1	100.8 (3)	C14—C24—H24C	109.5
C12—C2—C1	116.0 (4)	H24A—C24—H24C	109.5
C11—C2—C1	114.2 (3)	H24B—C24—H24C	109.5
N1—C3—N2	108.4 (4)		
			
O3—Ho1—O1—N1	79.9 (4)	O5—Ho1—N6—C16	123.4 (3)
O9—Ho1—O1—N1	−110.1 (3)	N3—Ho1—N6—C16	−105.7 (3)
O6—Ho1—O1—N1	−175.7 (3)	Ho1—O5—N7—O7	178.9 (4)
O8—Ho1—O1—N1	−87.8 (3)	Ho1—O5—N7—O6	−1.2 (4)
O11—Ho1—O1—N1	−6.0 (3)	Ho1—O6—N7—O7	−178.9 (3)
O12—Ho1—O1—N1	−33.4 (3)	Ho1—O6—N7—O5	1.2 (4)
O5—Ho1—O1—N1	176.7 (3)	Ho1—O9—N8—O10	−175.8 (5)
N3—Ho1—O1—N1	46.6 (3)	Ho1—O9—N8—O8	3.3 (4)
N6—Ho1—O1—N1	113.3 (3)	Ho1—O8—N8—O10	175.8 (5)
O1—Ho1—O3—N4	81.5 (3)	Ho1—O8—N8—O9	−3.3 (4)
O9—Ho1—O3—N4	−86.0 (3)	Ho1—O11—N9—O13	−169.4 (3)
O6—Ho1—O3—N4	−32.7 (3)	Ho1—O11—N9—O12	10.2 (3)
O8—Ho1—O3—N4	−109.0 (3)	Ho1—O12—N9—O13	169.6 (3)
O11—Ho1—O3—N4	−179.7 (3)	Ho1—O12—N9—O11	−10.0 (3)
O12—Ho1—O3—N4	−173.9 (2)	O1—N1—C1—C9	−47.7 (4)
O5—Ho1—O3—N4	−4.0 (3)	C3—N1—C1—C9	141.2 (4)
N3—Ho1—O3—N4	112.5 (3)	O1—N1—C1—C10	72.5 (4)
N6—Ho1—O3—N4	45.4 (3)	C3—N1—C1—C10	−98.6 (4)
O1—Ho1—O5—N7	171.9 (3)	O1—N1—C1—C2	−169.2 (3)
O3—Ho1—O5—N7	−35.1 (3)	C3—N1—C1—C2	19.7 (4)
O9—Ho1—O5—N7	89.5 (2)	O2—N2—C2—C12	−44.4 (5)
O6—Ho1—O5—N7	0.7 (2)	O2—N2—C2—C12	−44.4 (5)
O8—Ho1—O5—N7	53.1 (3)	C3—N2—C2—C12	147.2 (4)
O12—Ho1—O5—N7	132.9 (2)	O2—N2—C2—C11	73.5 (5)
N3—Ho1—O5—N7	−136.2 (2)	O2—N2—C2—C11	73.5 (5)
N6—Ho1—O5—N7	−85.7 (3)	C3—N2—C2—C11	−94.9 (4)
O1—Ho1—O6—N7	−9.6 (3)	O2—N2—C2—C1	−167.7 (4)
O3—Ho1—O6—N7	145.3 (3)	O2—N2—C2—C1	−167.7 (4)
O9—Ho1—O6—N7	−74.6 (2)	C3—N2—C2—C1	23.8 (4)
O8—Ho1—O6—N7	−128.9 (3)	N1—C1—C2—N2	−23.9 (3)
O11—Ho1—O6—N7	−178.5 (2)	C9—C1—C2—N2	−141.5 (4)
O12—Ho1—O6—N7	−108.7 (2)	C10—C1—C2—N2	88.8 (4)
O5—Ho1—O6—N7	−0.7 (2)	N1—C1—C2—C12	−143.7 (4)
N3—Ho1—O6—N7	80.6 (3)	C9—C1—C2—C12	98.8 (5)
N6—Ho1—O6—N7	72.4 (2)	C10—C1—C2—C12	−30.9 (6)
O1—Ho1—O8—N8	−25.7 (3)	N1—C1—C2—C11	87.7 (4)
O3—Ho1—O8—N8	159.5 (3)	C9—C1—C2—C11	−29.8 (5)
O9—Ho1—O8—N8	1.9 (2)	C10—C1—C2—C11	−159.5 (4)
O6—Ho1—O8—N8	86.3 (3)	O1—N1—C3—N2	−176.1 (3)
O11—Ho1—O8—N8	−135.9 (3)	C1—N1—C3—N2	−5.5 (4)
O12—Ho1—O8—N8	−81.2 (3)	O1—N1—C3—C4	8.9 (6)
O5—Ho1—O8—N8	46.3 (3)	C1—N1—C3—C4	179.5 (3)
N3—Ho1—O8—N8	−121.2 (3)	O2—N2—C3—N1	179.6 (4)
N6—Ho1—O8—N8	119.6 (3)	O2—N2—C3—N1	179.6 (4)
O1—Ho1—O9—N8	154.3 (3)	C2—N2—C3—N1	−12.5 (4)
O3—Ho1—O9—N8	−31.1 (3)	O2—N2—C3—C4	−5.3 (6)
O6—Ho1—O9—N8	−82.7 (3)	O2—N2—C3—C4	−5.3 (6)
O8—Ho1—O9—N8	−2.0 (2)	C2—N2—C3—C4	162.6 (4)
O11—Ho1—O9—N8	39.6 (3)	C8—N3—C4—C5	−4.3 (5)
O12—Ho1—O9—N8	76.9 (3)	Ho1—N3—C4—C5	163.1 (3)
O5—Ho1—O9—N8	−136.3 (3)	C8—N3—C4—C3	171.7 (3)
N3—Ho1—O9—N8	120.7 (3)	Ho1—N3—C4—C3	−20.8 (5)
N6—Ho1—O9—N8	−130.0 (3)	N1—C3—C4—N3	30.2 (6)
O1—Ho1—O11—N9	−40.5 (2)	N2—C3—C4—N3	−144.0 (4)
O3—Ho1—O11—N9	167.2 (2)	N1—C3—C4—C5	−153.7 (4)
O9—Ho1—O11—N9	42.2 (2)	N2—C3—C4—C5	32.1 (6)
O6—Ho1—O11—N9	128.3 (2)	N3—C4—C5—C6	2.9 (6)
O8—Ho1—O11—N9	76.5 (2)	C3—C4—C5—C6	−173.0 (4)
O12—Ho1—O11—N9	−5.9 (2)	C4—C5—C6—C7	0.6 (7)
N3—Ho1—O11—N9	−93.0 (2)	C5—C6—C7—C8	−2.3 (7)
N6—Ho1—O11—N9	−145.9 (2)	C4—N3—C8—C7	2.5 (6)
O1—Ho1—O12—N9	154.3 (2)	Ho1—N3—C8—C7	−167.1 (3)
O3—Ho1—O12—N9	−1.0 (2)	C6—C7—C8—N3	0.8 (7)
O9—Ho1—O12—N9	−127.0 (2)	O3—N4—C13—C21	−50.9 (4)
O6—Ho1—O12—N9	−92.4 (3)	C15—N4—C13—C21	135.0 (4)
O8—Ho1—O12—N9	−71.8 (2)	O3—N4—C13—C22	67.7 (4)
O11—Ho1—O12—N9	5.8 (2)	C15—N4—C13—C22	−106.5 (4)
O5—Ho1—O12—N9	−168.90 (19)	O3—N4—C13—C14	−173.0 (3)
N3—Ho1—O12—N9	81.6 (2)	C15—N4—C13—C14	12.9 (4)
N6—Ho1—O12—N9	85.9 (3)	O4—N5—C14—C24	−51.0 (5)
Ho1—O1—N1—C3	−60.2 (4)	O4—N5—C14—C24	−51.0 (5)
Ho1—O1—N1—C1	129.9 (3)	C15—N5—C14—C24	136.9 (4)
O2—O2—N2—C3	0.0 (5)	O4—N5—C14—C23	68.0 (5)
O2—O2—N2—C2	0.0 (5)	O4—N5—C14—C23	68.0 (5)
O1—Ho1—N3—C8	159.2 (3)	C15—N5—C14—C23	−104.0 (4)
O3—Ho1—N3—C8	−7.6 (3)	O4—N5—C14—C13	−173.4 (4)
O9—Ho1—N3—C8	−166.1 (2)	O4—N5—C14—C13	−173.4 (4)
O6—Ho1—N3—C8	51.4 (3)	C15—N5—C14—C13	14.6 (4)
O8—Ho1—N3—C8	−83.9 (3)	N4—C13—C14—N5	−14.9 (4)
O11—Ho1—N3—C8	−69.2 (3)	C21—C13—C14—N5	−130.9 (4)
O12—Ho1—N3—C8	−122.9 (3)	C22—C13—C14—N5	98.5 (4)
O5—Ho1—N3—C8	109.3 (3)	N4—C13—C14—C24	−133.6 (3)
N6—Ho1—N3—C8	59.8 (3)	C21—C13—C14—C24	110.3 (4)
O1—Ho1—N3—C4	−8.7 (3)	C22—C13—C14—C24	−20.3 (5)
O3—Ho1—N3—C4	−175.5 (3)	N4—C13—C14—C23	98.1 (4)
O9—Ho1—N3—C4	26.1 (4)	C21—C13—C14—C23	−17.9 (5)
O6—Ho1—N3—C4	−116.4 (3)	C22—C13—C14—C23	−148.5 (4)
O8—Ho1—N3—C4	108.2 (3)	O3—N4—C15—N5	−178.0 (3)
O11—Ho1—N3—C4	122.9 (3)	C13—N4—C15—N5	−4.3 (5)
O12—Ho1—N3—C4	69.3 (3)	O3—N4—C15—C16	3.1 (6)
O5—Ho1—N3—C4	−58.6 (3)	C13—N4—C15—C16	176.8 (4)
N6—Ho1—N3—C4	−108.0 (3)	O4—N5—C15—N4	−178.7 (4)
Ho1—O3—N4—C15	−55.6 (5)	O4—N5—C15—N4	−178.7 (4)
Ho1—O3—N4—C13	131.0 (3)	C14—N5—C15—N4	−7.1 (5)
O4—O4—N5—C15	0.0 (3)	O4—N5—C15—C16	0.2 (7)
O4—O4—N5—C14	0.0 (2)	O4—N5—C15—C16	0.2 (7)
O1—Ho1—N6—C20	−10.9 (3)	C14—N5—C15—C16	171.8 (4)
O3—Ho1—N6—C20	154.9 (3)	C20—N6—C16—C17	−4.2 (6)
O9—Ho1—N6—C20	−80.3 (3)	Ho1—N6—C16—C17	157.7 (3)
O6—Ho1—N6—C20	−128.7 (3)	C20—N6—C16—C15	175.8 (3)
O8—Ho1—N6—C20	−162.3 (2)	Ho1—N6—C16—C15	−22.3 (5)
O11—Ho1—N6—C20	110.6 (3)	N4—C15—C16—N6	34.2 (6)
O12—Ho1—N6—C20	52.4 (3)	N5—C15—C16—N6	−144.6 (4)
O5—Ho1—N6—C20	−73.9 (3)	N4—C15—C16—C17	−145.8 (4)
N3—Ho1—N6—C20	56.9 (3)	N5—C15—C16—C17	35.4 (6)
O1—Ho1—N6—C16	−173.5 (3)	N6—C16—C17—C18	0.3 (6)
O3—Ho1—N6—C16	−7.7 (3)	C15—C16—C17—C18	−179.6 (4)
O9—Ho1—N6—C16	117.1 (3)	C16—C17—C18—C19	3.6 (7)
O6—Ho1—N6—C16	68.7 (3)	C17—C18—C19—C20	−3.5 (7)
O8—Ho1—N6—C16	35.0 (4)	C16—N6—C20—C19	4.3 (6)
O11—Ho1—N6—C16	−52.1 (3)	Ho1—N6—C20—C19	−160.6 (3)
O12—Ho1—N6—C16	−110.2 (3)	C18—C19—C20—N6	−0.5 (7)

### Hydrogen-bond geometry (Å, º)

**Table:**

*D*—H···*A*	*D*—H	H···*A*	*D*···*A*	*D*—H···*A*
C8—H8···O11^i^	0.93	2.42	3.053 (5)	126
C11—H11*B*···O13^ii^	0.96	2.54	3.460 (6)	161
C18—H18···O4^iii^	0.93	2.56	3.450 (6)	160
C20—H20···O7^iv^	0.93	2.37	3.193 (5)	147
C22—H22*C*···O13^i^	0.96	2.53	3.239 (6)	131
C24—H24*B*···O10^v^	0.96	2.40	3.324 (6)	162

Symmetry codes: (i) −*x*, −*y*+1, −*z*; (ii) −*x*+1, −*y*+1, −*z*; (iii) −*x*−1/2, *y*−1/2, −*z*+1/2; (iv) −*x*+1/2, *y*−1/2, −*z*+1/2; (v) −*x*, −*y*+2, −*z*.
